# A Case of Synchronous Bilateral Canalicular Adenoma and Polymorphous Adenocarcinoma of the Minor Salivary Glands of the Upper Lip

**DOI:** 10.7759/cureus.87172

**Published:** 2025-07-02

**Authors:** Holly Boyes, Rahail Kumar, Mathew Alemkunnapuzha, Mohammed Anabtawi

**Affiliations:** 1 Oral and Maxillofacial Surgery, Pinderfields General Hospital, Wakefield, GBR; 2 Oral and Maxillofacial Surgery, Leeds General Infirmary, Leeds, GBR; 3 Histopathology, Pinderfields General Hospital, Wakefield, GBR

**Keywords:** canalicular adenoma, histopathology, oral and maxillofacial surgery, oral cavity, polymorphous adenocarcinoma, salivary gland

## Abstract

Multiple salivary gland tumours can occur in both major and minor salivary glands. Its histology can be greatly varied and include pleomorphic adenoma, canalicular adenoma, mucoepidermoid carcinoma, or polymorphous adenocarcinoma, amongst other pathologies.

A 76-year-old lady was referred on a two-week-wait pathway to our oral and maxillofacial surgery department complaining of a one-year history of painless lumps in her upper lip, which were gradually increasing in size. Examination in the clinic revealed four distinct lumps in the upper lip that were firm on palpation but mobile. Histopathological examination revealed the two medial lesions to be polymorphous adenocarcinomas and the two lateral lesions to be canalicular adenomas.

Whilst our case represents uncommon pathologies, our patient’s demographics are typical of others in the literature. This highlights the importance of adequate biopsies to gain a representative sample, as well as awareness that multiple salivary gland pathologies can synchronously exist.

## Introduction

Salivary gland neoplasms represent a diverse group of tumours that can occur in both major and minor salivary glands [1]. They account for less than 5% of all head and neck neoplasms, and while most arise from the major salivary glands, approximately 10-25% are of minor salivary gland origin, commonly located in the palate, upper lip, and buccal mucosa [2,3]. Though these tumours may occur as isolated lesions, multiple synchronous tumours, especially involving both benign and malignant histology, are notably uncommon.

Tumours of the salivary glands may present with a broad spectrum of histopathological features, ranging from benign entities such as pleomorphic adenoma and canalicular adenoma to malignant forms including mucoepidermoid carcinoma, adenoid cystic carcinoma, and polymorphous adenocarcinoma [3, 4].

Most reported cases of multiple tumours occur in the parotid gland, with synchronous benign and malignant lesions in minor salivary glands being exceedingly rare. A limited number of reports have documented such dual pathologies in the upper lip, including canalicular adenoma and polymorphous adenocarcinoma occurring concurrently [1,5,6].

We present a rare case of bilateral upper lip canalicular adenomas occurring synchronously with polymorphous adenocarcinomas. This case serves to underscore the diagnostic complexity and necessity of complete histological evaluation of all lesions, particularly when multiple lesions are present.

## Case presentation

A 76-year-old lady was referred by her dentist on a two-week wait pathway to our oral and maxillofacial surgery department, complaining of a one-year history of painless lumps in her upper lip, which were gradually increasing in size. She denied any bleeding, paraesthesia, taste disturbance, dry mouth, or constitutional symptoms.

Her medical history includes chronic obstructive pulmonary disease, hypertension, osteoarthritis, hypothyroidism, type 2 diabetes mellitus, hypercholesterolaemia, and cataracts. She has an allergy to nuts. Her social history revealed she is an ex-smoker, having stopped at age 74, and a non-drinker.

Examination in the clinic revealed four distinct lumps in the upper lip that were firm on palpation but mobile: (A) in the UR4 region, measuring 8 mm in diameter; (B) in the UR2 region, 4 mm; (C) in the UL2 region, 5 mm; and (D) in the UL4 region, 7 mm. There was no cervical lymphadenopathy, and was edentulous in the upper and lower jaws.

**Figure 1:**
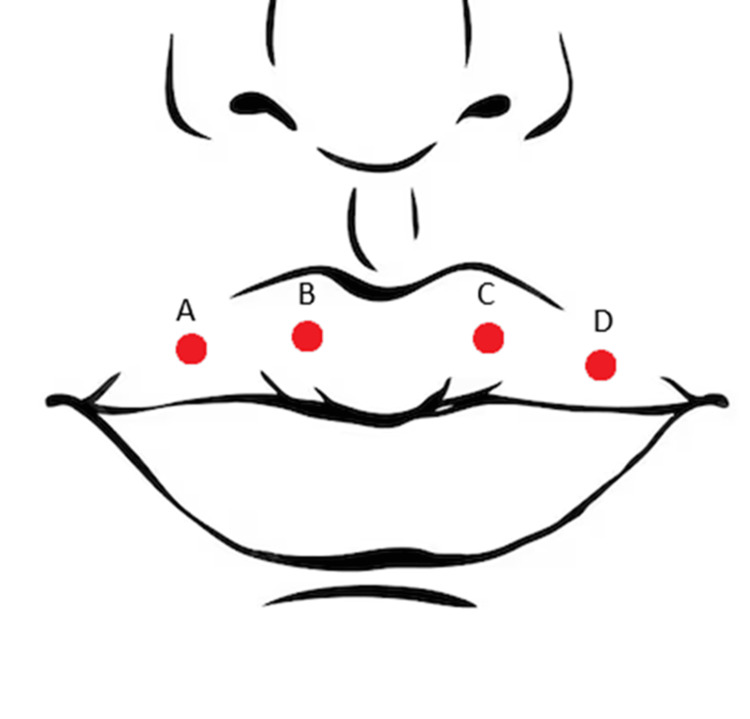
Schematic illustrating clinical locations of lesions

Histopathological examination revealed the following diagnoses (see Figure 1): (A) canalicular adenoma, (B) polymorphous adenocarcinoma of the minor salivary gland, (C) polymorphous adenocarcinoma of the minor salivary gland, and (D) canalicular adenoma.

Following histopathological assessment, she was referred to the regional head and neck cancer service for further investigation and management. An MRI of the neck showed no evidence of nodal disease. She also underwent a staging CT of the chest, which revealed several lung nodules of varying sizes. Her case was discussed at the head and neck multi-disciplinary team (MDT) meeting, and was listed for wide local excision and local reconstruction of the adenocarcinomatous lesions in her upper lip, along with a referral to the local lung MDT regarding her lung nodules.

She underwent wide local excision (5 mm margins) and primary closure of the adenocarcinomatous lip lesions under general anaesthesia, from which she recovered well with a minimal amount of residual numbness. The histopathology showed complete excision of both polymorphous adenocarcinoma (pT1NXM0), and she was scheduled for a routine follow-up.

**Figure 2:**
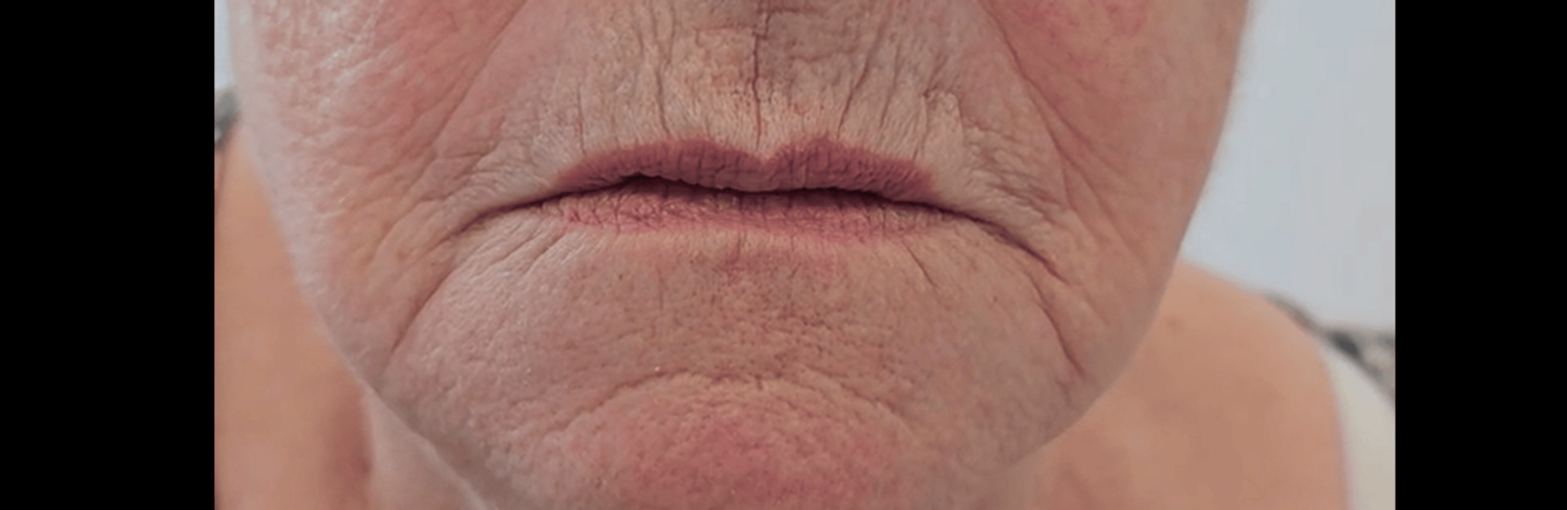
Post-operative lips at rest

**Figure 3:**
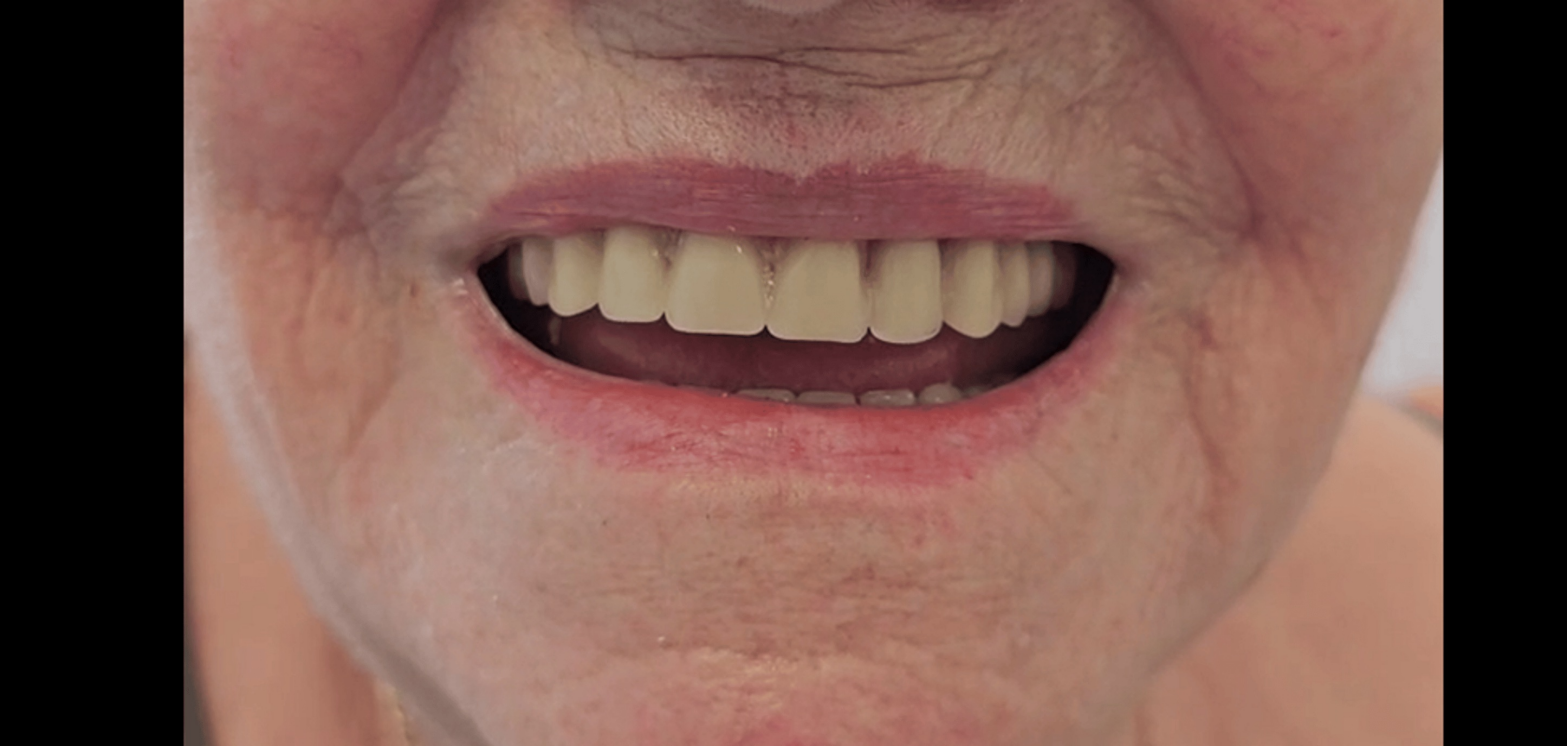
Post-operative lips smiling

**Figure 4:**
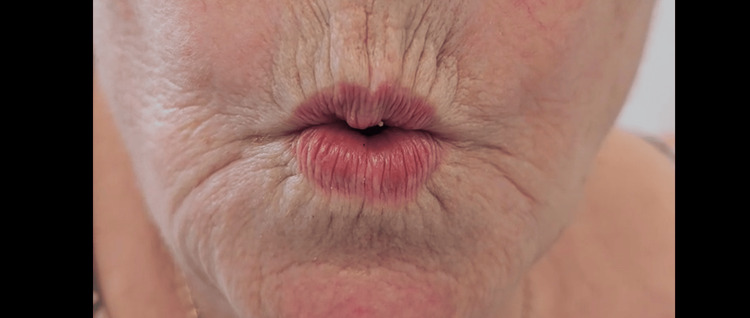
Post-operative lips pursed

The lung MDT recommended surveillance CT scans, which showed no concerning findings on follow up and she was discharged from the respiratory department after four months. She was seen by the oral and maxillofacial surgery team every six weeks for a year following her procedure, with four-monthly neck ultrasound scans (all of which were negative for disease). She is currently on four-monthly clinic reviews and progressing well (Figures 2-4).

## Discussion

Minor salivary gland tumours present a diagnostic challenge due to their varied histopathology and potential for multiple synchronous lesions. One study analysed 213 cases of minor salivary gland tumours and found 56% were benign tumours (most commonly pleomorphic adenoma, followed by canalicular adenoma) and 44% were malignant (most commonly mucoepidermoid carcinoma, followed by adenoid cystic carcinoma, then polymorphous low-grade adenocarcinoma) [3].

Canalicular adenoma

Canalicular adenomas are benign salivary gland tumours that often present as a slow-growing nodule in the upper lip, more commonly found in women over the age of 50 [7]. They are well-circumscribed and rarely recur after excision. Although typically solitary, multifocal and bilateral presentations have been documented [8-10]. They arise from the terminal ducts of minor salivary glands, are encapsulated, and characterised histologically by columnar or cuboidal cells in a loose, highly vascular stroma [7].

Polymorphous adenocarcinoma

Polymorphous adenocarcinoma most commonly occurs in minor salivary glands of the palate [11]. Morphologically, it can exhibit a variety of growth patterns, including solid, cribriform, tubular, trabecular, fascicular, and papillary patterns [12, 13]. It frequently presents as an asymptomatic, slow-growing mass; it is more commonly seen in women, with a peak incidence in the sixth to seventh decade of life. It commonly presents in the palate; it may be locally destructive and often displays perineural invasion, though distant metastases are rare. Microscopically, the tumour is unencapsulated, infiltrative, with cells arranged in diverse architectural patterns [14].

Clinical significance

Most reported cases of synchronous tumours involve the major glands, especially the parotid [15]. In the literature, only a few cases describe synchronous canalicular adenoma and polymorphous adenocarcinoma, with very limited cases involving bilateral lesions [1, 5, 6]. From a clinical perspective, the identification of one benign lesion should not preclude investigation of adjacent or additional lesions, as these may harbour malignant pathology. This case exemplifies the need for individualised assessment and histopathologic confirmation of each lesion. The patient’s age, sex, and anatomical location align with typical presentations of both canalicular adenoma and polymorphous adenocarcinoma.

## Conclusions

Though each tumour entity individually aligns with the known epidemiological and clinical patterns, their concurrent appearance underscores the necessity of detailed clinical, radiological, and pathological assessment. Multiple biopsies should be obtained in cases of multifocal lesions to ensure accurate diagnosis and treatment planning. Clinicians should maintain a high index of suspicion when encountering multiple upper lip lesions, particularly in elderly female patients, as synchronous salivary gland tumours, though rare, carry important diagnostic and management implications.
